# A field study to explore user experiences with socially assistive robots for older adults: emphasizing the need for more interactivity and personalisation

**DOI:** 10.3389/frobt.2025.1537272

**Published:** 2025-03-28

**Authors:** Bob M. Hofstede, Sima Ipakchian Askari, Dirk Lukkien, Laëtitia Gosetto, Janna W. Alberts, Ephrem Tesfay, Minke ter Stal, Tom van Hoesel, Raymond H. Cuijpers, Martijn H. Vastenburg, Roberta Bevilacqua, Giulio Amabili, Arianna Margaritini, Marco Benadduci, Julie Guebey, Mohamed Amine Trabelsi, Ilaria Ciuffreda, Sara Casaccia, Wijnand IJsselsteijn, Gian Marco Revel, Henk Herman Nap

**Affiliations:** ^1^ Vilans Centre of Expertise for Long-Term Care, Utrecht, Netherlands; ^2^ Human-Technology Interaction Group, Department of Industrial Engineering and Innovation Sciences, Eindhoven University of Technology, Eindhoven, Netherlands; ^3^ Copernicus Institute of Sustainable Development, Utrecht University, Utrecht, Netherlands; ^4^ EvaLab, Division of Medical Information Science (SIMED), University Hospitals of Geneva (HUG), Geneva, Switzerland; ^5^ ConnectedCare Services b.v., Arnhem, Netherlands; ^6^ IRCCS INRCA, Scientific Direction, Ancona, Italy; ^7^ Dipartimento di Ingegneria Industriale e Scienze Matematiche, Università Politecnica delle Marche, Ancona, Italy

**Keywords:** socially assistive robots, evaluation studies, interactivity, personalisation, older adults, care network, gerontechnology, home environment

## Abstract

Older adults often desire to remain in their homes for as long as possible, and Socially Assistive Robots (SARs) can play a role in supporting this goal. However, the acceptance and adoption rates of SARs remain relatively low, suggesting that current designs may not fully address all user needs. Field studies in Human-Robot Interaction, particularly those involving multiple end-users, remain limited. Nevertheless, such studies are crucial for identifying factors that shape the user experience with SARs, potentially improving their acceptance and adoption in healthcare settings. Therefore, this study aims to explore user perspectives, referred to as factors, that could guide design considerations for SAR development. We conducted a field study with 90 participants across Italy, Switzerland, and the Netherlands to identify these factors and their implications for improving the SAR user experience for older adults and their formal and informal caregivers. SARs were placed in the homes of older adults, and interviews were conducted with the three groups of primary end-users, at the beginning, midpoint, and end of the two-to six-week trial period. We initially focused on four factors (personalisation, interactivity, embodiment, and ethical considerations), identified in earlier design phases of the related 3-year Guardian project. Our findings confirmed the importance of these factors while uncovering additional ones. Personalisation and interactivity emerged as the most important ones among these factors. Based on our insights, we recommend involving all primary end-users in SAR research and design process and prioritising field studies to refine design elements. In conclusion, our study identified six factors for SAR design that can enhance the user experience: personalisation, interactivity, embodiment, ethical considerations, connectedness, and dignity. These findings provide valuable guidance for developing SARs that may better address the needs of older adults and their caregivers.

## 1 Introduction

Most older adults in the US want to live in their own homes as long as possible (Binette and Farago, 2021). A similar trend was found in the Netherlands (Centraal Bureau voor de Statistiek, 2020), and this desire is expected to grow globally in upcoming years (Rogers and Mitzner, 2017). At the same time, healthcare systems face growing pressure due to the double-ageing society, where the number of older adults is increasing while the healthcare workforce is also ageing and retiring (World Health Organization, 2022). In response to such desires of older adults and the challenges posed by ageing societies, many countries increasingly focus on supporting home-based care, including the integration of intelligent gerontechnologies (Ienca et al., 2017). Gerontechnologies are technologies developed using insights from the interdisciplinary field of gerontology, aimed to support successful ageing (Fozard et al., 2000). Examples include telemedicine, remote monitoring, fall detection systems, medication dispensers, (socially) assistive robots, and smart home technologies.

Furthermore, care is often fragmented among various healthcare providers, which can reduce its effectiveness Robben et al. (2012). Studies indicate that gerontechnologies can help reduce this fragmentation by improving communication between formal caregivers (i.e., professional caregivers), informal caregivers (i.e., mostly family caregivers or other acquaintances), and older adults (Bodenheimer et al., 2002; De Haes, 2006). For example, Kruse et al. (2018) found that allowing caregivers to register clients’ health statuses online can enhance communication between healthcare providers which resulted in more quality and efficiency of care. Thus, gerontechnologies have the opportunity to support the desire of older adults to remain in their own homes as long as possible, and diminish the negative effects of fragmented care.

Socially assistive robots (SARs) have been found to support older adults in home-based care and have great potential in healthcare (Gasteiger et al., 2021). SARs can be defined as socially intelligent agents (Dautenhahn and Billard, 1999) often featuring (virtual) embodiment (Vandemeulebroucke et al., 2020) that provide assistance to users through social interaction. In care for older adults, a SAR can be seen as an intelligent telehealth application (i.e., offering care from a distance and over the internet) as was argued by McLendon (2000). Unlike robots focused on physical tasks, SARs assist through social interaction, often providing daytime structure (e.g., reminders for medicine and food intake or activity suggestions) and companionship (Feil-Seifer and Mataric, 2005; Bouwhuis, 2016; Vercelli et al., 2018). In the study by Tanaka et al. (2012) a communication robot was developed for frail older adults, which showed promising results. After using the robot for 8 weeks, 34 older female participants with dementia had greater improvements in their cognitive functions and sleep quality, compared to a control group. Altogether, SARs have the potential to address societal challenges and fulfil needs in healthcare for older adults (Fan et al., 2021; Gutman et al., 2018; Pino et al., 2015; Gasteiger et al., 2022).

Although various promising SARs have been developed and introduced to the market, research indicates that their adoption by intended users remains limited, with successful implementation being a central problem that many developers face (Greenhalgh et al., 2017). A reason may be that SARs are not yet sufficiently aligned with user needs. For instance, while SAR acceptance improved among healthcare personnel after the COVID-19 pandemic (Getson and Nejat, 2022), this does not necessarily result in increased acceptance for other types of end-users (i.e., older adults and informal caregivers) as was concluded by Merkel and Kucharski (2019). Furthermore, acceptance levels differ across these groups of end-users. For instance, older adults are more receptive when the benefits of a SAR to them are clear (Di Nuovo et al., 2018; Melenhorst et al., 2006), and the acceptance of informal caregivers varies based on factors like age, gender, and education levels (Wójcik et al., 2021). Hence, user needs vary among different end-users of SARs in healthcare, making it essential to conduct research involving all user groups.

In addition, as argued by Cortellessa et al. (2021) and Gasteiger et al. (2021), most SAR research takes place in controlled settings (i.e., labstudies), resulting in limited real-world context in Human-Robot Interaction (HRI) studies. Limited real-world testing can lead to inaccurate expectations and can affect user acceptance and experience negatively (De Graaf et al., 2019). Therefore, more field studies are recommended to enhance the external validity of HRI research (Gasteiger et al., 2021). Such field studies can either translate findings from the lab to real-world settings or focus directly on the development and implementation of solutions in practice. Conducting research *in situ*, meaning within real-world environments, further enhances external validity by capturing the complexities of everyday contexts (Winkle et al., 2020), a finding that was also concluded in more recent research by Cooper et al. (2023).

Although the number of robotic field studies has increased in recent years, the number of field studies that have been conducted with SARs for older adults remains limited. Examples include, the ENRICHME robot that was tested in the homes of older adults in three European pilot sites and proved to be an effective assistant (Coşar et al., 2020), or the Hobbit robot which aimed to support the independent living of older adults, which was also tested at the homes of older adults in three European pilot sites (Fischinger et al., 2016). Furthermore, the study by Gross et al. (2019) showed that older adults could better shape their everyday lives at home because of a mobile companion robot that provided them reminders and support. Additionally, three studies by Luperto and colleagues report results of robotic solutions that were tested in field studies in the homes of older adults, and report positive findings on the integration of robots with other smart systems (Luperto et al., 2022; Luperto et al., 2023a; Luperto et al., 2023b). Despite these examples, field studies in HRI remain limited, especially those involving more than one of the three SAR primary end user groups in healthcare (older adults, formal caregivers, and informal caregivers) and focused on socially assistive robots. However, conducting such comprehensive field studies can reveal factors influencing user experience, which can provide input for design considerations aimed at improving SARs to better address diverse user needs (Kujala, 2002) and increase effectiveness by taking into account user experiences of all three primary user groups which are dependent actors in the care and health field (Robben et al., 2012; Suijkerbuijk et al., 2019).

In the following paragraphs, we present four factors identified through fieldwork of a 3-year project involving the iterative development of a SAR. The project included usability testing in the first year (Ciuffreda et al., 2023a), alpha testing through field studies in the second year (Amabili et al., 2024), and a summative evaluation in the third year. The four factors - personalisation, interactivity, embodiment, and ethical considerations - were primarily identified during the alpha testing phase. The factors form the basis of the summative evaluation presented in this paper.

According to Dautenhahn (1998) the concept of personalisation is defined as the attribute of a machine to adapt to the preferences of its end-user(s). In the context of Human-Robot Interaction (HRI), Gasteiger et al. (2021) add that a robot should meet the personal needs or preferences of each end-user, and hence, should be adaptive to the context of use. Research conducted by Sebri and Savioni (2020) has shown that the perceived value (i.e., usefulness) of gerontechnologies, including SARs, is strongly related to the level of customisation (i.e., the possibility of customers to personalise a product). In line with these findings, Shryock and Meeks (2022) have found that interventions proposing tailored activities, based on personality and preference, are more effective in consistently supporting individuals with cognitive impairments than non-tailored interventions. Other examples include enabling end-users to customize communication-related variables of the SAR (Hofstede et al., 2022). However, since these findings were obtained in laboratory settings, conducting field studies would provide valuable insights, as advocated by Cortellessa et al. (2021). Evaluating the user experience of customizing the SAR’s voice gender, volume, and speaking rate, as suggested in the lab study by Hofstede et al. (2022), could reveal valuable insights. Additionally, researchers might manually personalize the SAR’s spoken content based on the end-user’s personal details, as recommended by Kaptein et al. (2012), Hofstede et al. (2022) and Shryock and Meeks (2022).

Interactivity is another factor that can influence the user experience with SARs. Xue et al. (2021) found that proactive interaction behaviour by robots can increase user acceptance and satisfaction. Interactivity between robots and humans can be enriched through various actions carried out by the robot, including increased verbal communication using speech (Grigore et al., 2016) or through the incorporation of sounds responsive to touch, known as vocables (Zhang and Fitter, 2023). Non-verbal communication can also play a pivotal role, involving bodily movements during the interaction or idle movements (Cuijpers and Knops, 2015). Augmenting interactivity (e.g., through meaningful motions) can increase the level of social verification and social responsiveness (Cuijpers and Knops, 2015). Social verification refers to individuals confirming to themselves that they are engaged in meaningful communication, while social responsiveness refers to people treating computers or other media as real social entities, as described in the media equation hypothesis by Reeves and Nass (1996). Positive social responses to robots, such as liking and trust, have been shown to improve robot acceptance (Ghazali et al., 2020). To increase social responsiveness and, consequently, the acceptance of SARs, it is recommended to enhance their interactivity. For example, incorporating vocables, as described by Ciuffreda et al. (2023b), can enrich interaction through auditory feedback. Similarly, adding idle movements, such as subtle hand or head motions during conversation, can make SARs appear more socially responsive and engaging.

A third factor focuses on the role of embodiment in SARs. Fasola and Matarić (2013) found that SARs used as coach for older adults were evaluated better in terms of enjoyment, helpfulness, and social attraction when they had physical embodiment compared to virtually embodied SARs. Moreover, Tanaka et al. (2014) found that physical embodiment enhanced social telepresence compared to virtually embodied SARs. However, virtually embodied SARs have the advantage of lower development costs. Furthermore, virtual SARs can be perceived, in some situations, as easier to operate, which decreases the perceived workload, as was found in the study of Baba et al. (2021) in which a customer service robot was studied. The impact of embodiment on SAR user experience has not been extensively studied, and existing research presents contradictory findings. To address this, further research is recommended, particularly through field studies that involve iterative SAR design.

Finally, a fourth factor influencing the SAR user experience is the integration of ethical considerations, grounded in the principles of responsible innovation (RI). According to Owen et al. (2013), RI extends beyond SARs and holds relevance for enhancing the development of all types of innovations. Involving RI in the R&D-process includes mitigating risks related to undermining people’s privacy, autonomy and self-determination, consequently, it requires researchers, users and other stakeholders to take a critical look at the social and ethical implications of new technologies (Lukkien et al., 2023). By integrating RI principles, the R&D process can foster greater acceptance of SARs among all stakeholders, as RI seeks to ensure more ethical, societally desirable outcomes in the design and implementation of innovative solutions like SARs (Lukkien et al., 2023). Examples of RI include raising awareness among project partners during development through individual and collective reflective exercises to discuss their perspectives on RI. It also involves better expectation management, such as clearly explaining to end-users the capabilities and limitations of devices during evaluative (field) studies. Additionally, increasing user autonomy, for instance by offering the option to mute the system, as was found a user need in earlier iterations of SAR design by Ciuffreda et al. (2023a), can further support the goals of RI.

As mentioned earlier, field studies in Human-Robot Interaction are limited, particularly those involving all three primary end-users: older adults and their formal and informal caregivers. However, such studies can reveal important factors that influence the user experience with SARs, potentially enhancing their acceptance and adoption in healthcare. Therefore, this study aims to reflect on user perspectives, referred to as factors, that could guide design considerations for SAR development. We focus on personalisation, interactivity, embodiment, and ethical considerations, while also exploring additional factors that may impact the SAR user experience. Accordingly, we present a case study involving fieldwork with the three primary end-users of SARs for older adults.

### 1.1 The Guardian project

The case study presented in this paper is part of the Guardian project, an international collaboration within the European AAL program (Active and Assistive Living - AAL Europe, 2020, Projectnumber: aal-2019-6-120-CP), aimed at developing a SAR to assist older adults and their caregivers. The project involved a consortium of care organizations, commercial firms, and research institutes from the Netherlands, Italy, and Switzerland. Potential end-users participated in iterative co-design activities to define the functional and non-functional requirements of the SAR. Furthermore, the project incorporated RI through workshops designed to raise awareness and promote its integration into R&D activities, aiming for ethical acceptability, societal desirability, and sustainability throughout the development process (Owen et al., 2013; Von Schomberg, 2013).

Over the 3-year project, the Guardian system was developed, consisting of a SAR linked to a senior app and a caregiver app. The senior app, displayed on a tablet next to the SAR, allowed older adults to respond to reminders and suggestions, such as “how are you feeling?” and provide self-assessments on wellbeing or sleep quality, and inform about the completion of suggested activities. Caregivers could use the caregiver app to set reminders, suggest activities, request self-reports, and communicate with each other via a chat function. The caregiver app also displayed older adults’ responses to activity suggestions, wellbeing or sleep quality queries, and medicine and food intake, so caregivers could monitor the older adults. The SAR was designed to vocalize the reminders and suggestions that were set by caregivers, initiating interaction with older adults by speaking directly to them. However, the SAR could not respond to incoming sounds or speech from its end users. Still, older adults could also initiate interaction with the SAR by either stroking its head or tapping on the tablet displaying the senior app. Hence, the number of interactions varied depending on the older adult and the number of reminders or suggestions entered into the caregiver app. However, there were at least three interactions daily to prompt meal intake reminders, with no upper limit on interactions.

The SAR was placed on a table next to the tablet with the senior app. While mobility was disabled, the SAR could still move its arms and tilt its head while talking and respond to touch by producing sounds (i.e., vocables). Furthermore, during the studies in the Netherlands, two different types of SARs were used to compare user experiences with different embodied SARs: one with physical embodiment and another with virtual embodiment.

## 2 Materials and methods

The case study presented in this paper is a field study of the Guardian system during its summative evaluation, conducted from September to December 2022, across three countries: Italy, Switzerland, and the Netherlands.

### 2.1 Participants

In total, 90 participants participated in the summative evaluation of the Guardian project. Each of the participating older adults formed a triad together with one of their informal caregivers and one of their formal caregivers. However, in some cases, couples of older adults participated together, sharing a single informal caregiver. As a result, the numbers are not evenly distributed, and not all triads add up to exactly 30 participants in each category.

Participants were recruited via the care networks of the involved project partners, leading to 30 participants per country. Inclusion criteria for the older adults were being aged 65 years or older and requiring care by a care organization. Furthermore, each participating older adult had to have at least one formal caregiver (a healthcare professional) and one informal caregiver (usually a family member). Collaborating care organisations assessed the older adults as needing care but still capable of participating in interviews, ensuring a certain level of cognitive ability. No specific inclusion criteria were established for formal and informal caregivers. Demographic data of the participants are presented in Table 1 in which the Netherlands is abbreviated by NL, Italy by IT, and Switzerland by CH.

**TABLE 1:** Demographic data of the participants.

End-user	Country	Sample size	Gender (F/M)	Age (in years) (mean ± SD)
Senior	NL	13	8/5	81.5 ( ± 5.4)
	IT	10	5/5	75.4 ( ± 5.8)
	CH	9	2/7	77 ( ± 10.9)
Informal caregiver	NL	6	5/1	53.5 ( ± 2.3)
	IT	10	5/5	47.6 ( ± 9.7)
	CH	10	6/4	53.2 ( ± 16.2)
Formal caregiver	NL	11	8/3	40.7 ( ± 12.8)
	IT	10	6/4	39.3 ( ± 13.0)
	CH	10	7/3	44.3 ( ± 14.2)

### 2.2 Materials

To perform the field studies for each triad, the Misty robot (Misty Robotics, 2019) was needed as well as a tablet containing the senior app that was developed during the project. Furthermore, the majority of the participants did not have access to a stable WiFi connection. This issue was solved by installing a MiFi router (i.e., a mobile hotspot) at the older adult’s home to connect the SAR and the tablet containing the senior app to the internet. The informal and formal caregivers were supposed to log in to the caregiver app via their own devices, such as a laptop, a mobile phone or a tablet. Furthermore, formal and informal caregivers were required to have an email address as they needed it for their credentials to log in to the caregiver app. Lastly, we used informed consent forms, as well as interview scripts covering topics such as usefulness, usability, sociability, privacy, feelings of control and trust, interactivity and embodiment (see Supplementary Material). Furthermore, during the interviews with older adults eleven 5- or 7-point Likert-scale questions were asked based on constructs of the system usability scale (Brooke, 2013), the perceived persuasiveness questionnaire (Lehto et al., 2012), the IMPACT survey (Shofmann, 2021) and the IBM questionnaire (Lewis, 1995), which assess factors like system usability, persuasiveness, and user satisfaction. All interview topics and questionnaire constructs were based on an earlier developed study protocol related to the 3-year Guardian project (Margaritini et al., 2022).

#### 2.2.1 The guardian system

**FIGURE 1:**
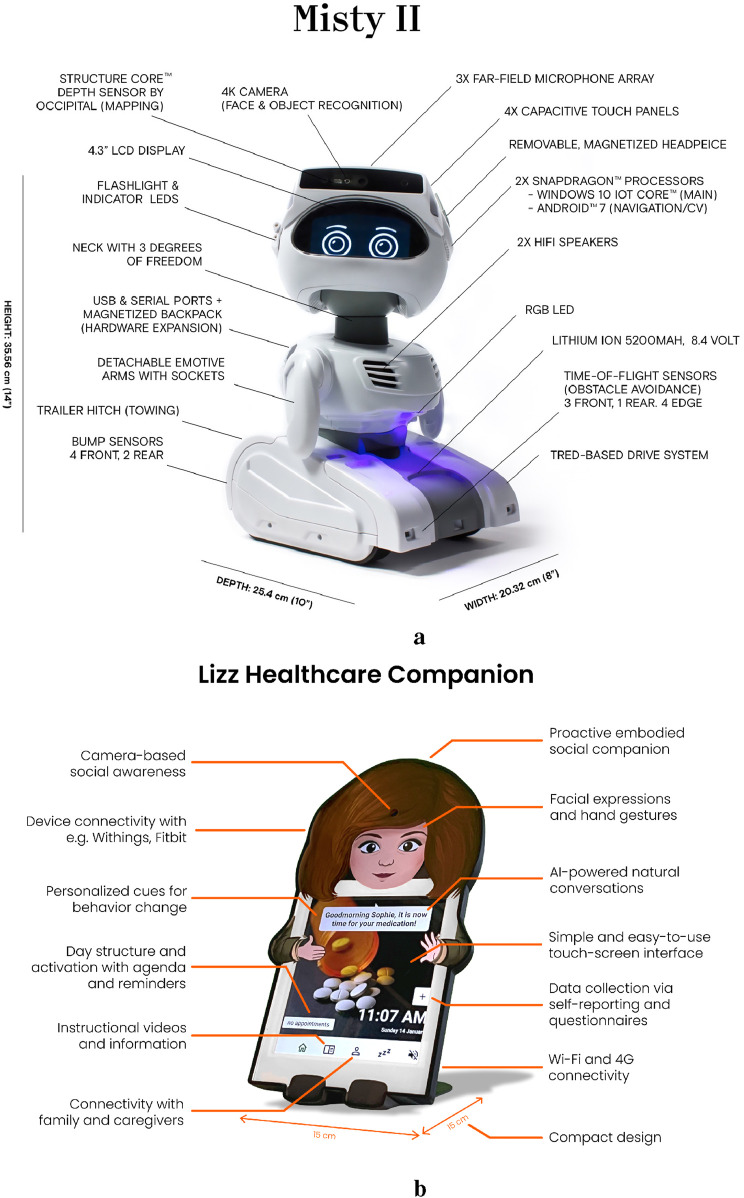
The two SARs being used during the summative evaluations of the Guardian Project **(A)** Misty II **(B)** Lizz. **(A)** Misty II from Misty Robotics (2019), reprinted from Business Wire. **(B)** Lizz - the digital healthcare assistant from (ConnectedCare, 2022, www.lizz.health).

The SAR was developed by Misty Robotics and is a tabletop robot with a screen on which eyes are projected (Misty Robotics, 2019). The robot can mobilise itself, but this functionality was disabled throughout the project to mitigate the risk of potentially hazardous situations that could, for example, lead to falls among older adults. Hence, the SAR was placed stationary at a central place in the living environment of the older adult, e.g., on a table. Furthermore, the Misty robot worked by programmed scripts of code, called skills, that determined the behaviour of the robot. Functions that were programmed in these skills involved the use of speech, tilting the head of the SAR, changing the eyes of the SAR, changing the colour of the LED on the SAR’s chest, responding to touch, moving the hands of the SAR, and looking at the user and making eye contact. The complete details of the SAR are shown in Figure 1A.

Compared to earlier field studies within this project (Ciuffreda et al., 2023a; Amabili et al., 2023), the Guardian prototype was improved for the summative evaluation in several ways. Increased interactivity was achieved by incorporating vocal responses to touch and the inclusion of idle movements, while personalization was enhanced through the ability to customize speech-related variables, and ethical considerations led to the inclusion of the ability to mute the system. In the Netherlands, personalized content in messages was introduced and the effects of embodiment were tested.

As indicated, during the 10 trials in the Netherlands, another type of SAR was used as well, called Lizz (Figure 1B), developed by one of the project partners (ConnectedCare, 2022). Both robots had similar functionalities. However, they differed in the layout of the applications (in terms of colors, fonts and font-sizes) and the design and embodiment of the robots. Lizz is a virtual robot shown on a tablet while Misty is a robot with a physical embodiment. The Misty robot was selected for its open platform, modularity, friendly appearance, and sensor capabilities, enabling easy programming and customization. Lizz was included to explore an alternative social robot with a different embodiment but with similar capabilities as Misty. All of the Guardian system’s components are shown in Table 2.

**TABLE 2:** The three components of the Guardian system.

Component	Used for	Primary end-user
SAR (Misty II and Lizz)	- Communication and companionship for older adult	Older adults
Senior application	- Establishing the connection between the caregiver application and the SAR - Responding to incoming reminders and suggestions - Submitting self-reports about sleep quality and wellbeing - Customizing the communication-related variables - Mute the complete system by putting it in “sleep mode”	Older adults
Caregiver application	- Setting reminders and suggestions at given time slots - Asking for wellbeing or sleep quality reports at given time slots - Sending direct messages, reminders or suggestions to older adults - Checking for incoming responses - Chatting with care network	Formal and informal caregivers

### 2.3 Procedure

**FIGURE 2:**
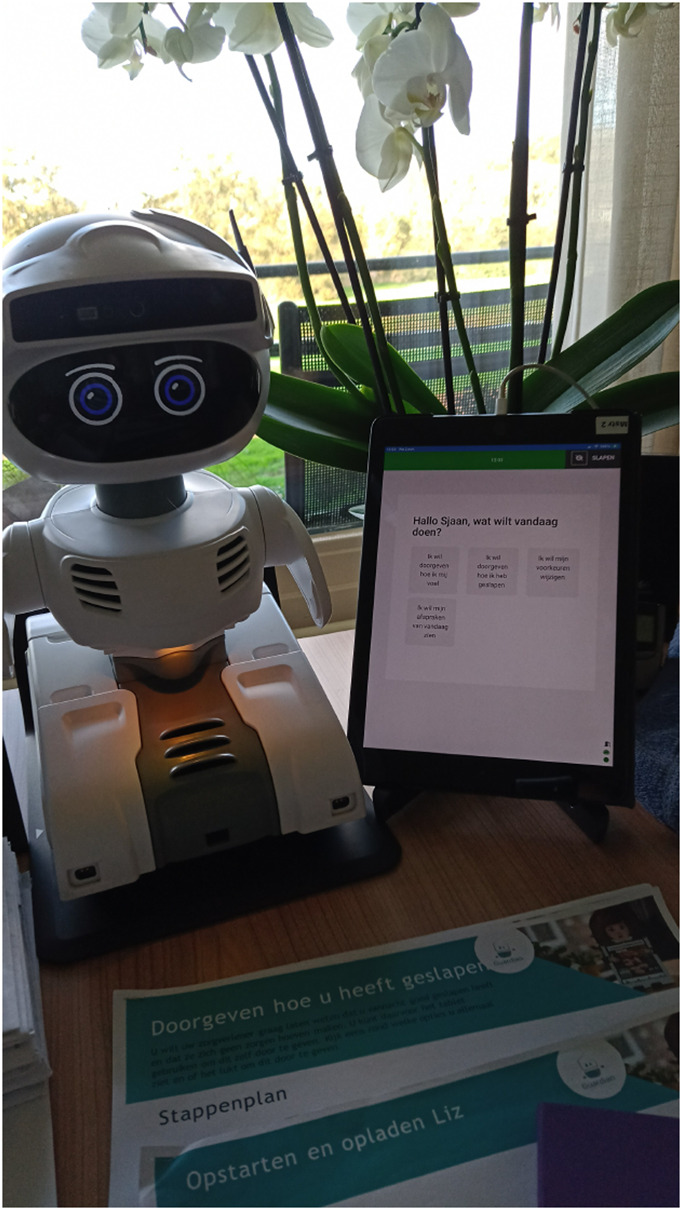
Setup of the Guardian system with the Misty robot.

All participants were grouped in triads, consisting of an older adult and one formal and informal caregiver, hereinafter called a triad. At the start of each trial, participants were asked to sign informed consent. Subsequently, the Guardian system was installed at the older adult’s home as depicted in Figure 2. Next, the first interview was completed and the researchers entered appointments and reminders into the system in consultation with the formal and informal caregivers. After 2 days, the researcher called the older adult to check whether any errors or malfunctions arose. In between and at the end of the test phase, the researchers completed the second and third interviews. Interviews with older adults were conducted in person, while interviews with caregivers were conducted either online via Microsoft Teams (Microsoft Corporation, 2016) or in person, depending on the participants’ convenience. Finally, at the end of the trial, the researchers uninstalled the system, removed it from the older adult’s home, and conducted a debriefing session with the participants. The exit strategy ensured that any remaining issues were addressed, and the data were securely archived for further analysis.

The field study protocols were designed with flexibility to accommodate country-specific challenges and optimize *in situ* testing within the available resources. This adaptability not only ensured alignment with the needs of participants and care organizations but also created unique opportunities for deeper exploration. For instance, extended testing periods and the evaluation of two distinct SARs were possible in some locations. An overview of the protocols for each country is provided in Table 3. In Italy, close collaboration with the client organization and strong participant engagement enabled a 6-week testing period, compared to the 2-week testing in the Netherlands and Switzerland. Furthermore, in the Netherlands, access to two robots facilitated a comparative study of Misty and Lizz, with each tested for 1 week, yielding insights into embodiment effects. Across all three countries, personalisation included customization options for volume, voice gender, and speaking rate. In the Netherlands, personalisation was further expanded by customizing message content based on participants’ personal interests, such as reminders about their favorite sports teams. In Switzerland, recruitment challenges were addressed by organizing three focus groups with the remaining nine participants to discuss the system once and gather feedback on the interview topics.

**TABLE 3:** Study protocols per country.

Country	Duration	Evaluated SAR(s)	Additional information
Italy	6 weeks	Misty	
Netherlands	2 weeks	Misty and Lizz	Both robots tested for 1 week each; Also evaluated personalisation on content level
Switzerland	2 weeks	Misty	A total of 21 participants took part in the field testing. Subsequently, three focus groups were organized for which a total of nine new participants were recruited (three triads of older adults and their formal and informal caregivers) due to recruitment challenges. Total N = 30

### 2.4 Data analysis

Data was collected through the aforementioned interviews containing demographic data and data from interviews at the start, intermediate and end of each trial. Hence, for each triad, data was collected from a total of nine interviews (three per person involved in the triad). During the interviews, researchers made notes. In order to avoid intrusiveness, no recordings or transcripts were made. All data was pseudonymised, and thematically analysed (Braun and Clarke, 2019) was conducted on the complete set of qualitative data. For the thematic analysis two researchers first coded the complete dataset. This resulted in a first set of themes, for example,: “older adults see the added value of medication and appointment reminders both before and after use”. In a second iteration, the same two researchers defined these themes in more detail, and sub-themes were constructed. For example,: “before and after using the Guardian system, older adults experienced added value of the medication reminders and activity suggestions” with as on accompanying subtheme: “timing and context are important determinants of the relevance of reminders and suggestions”.

Data from the eleven Likert-Scale questions among older adults were analysed in Excel (Microsoft Corporation, 2019) in a descriptive manner which involved discussing the mean, mode, median or range (Fisher and Marshall, 2009).

## 3 Results

This section will explain the themes and subthemes that have been identified through the thematic analysis based on the data of the summative evaluation, accompanied by participant quotes, and answers by older adults on the eleven Likert-Scale questions. A complete overview of answers from older adults to the eleven Likert-Scale questions is depicted in the Supplementary Material. Formal caregivers are abbreviated as FCs and informal caregivers are abbreviated as ICs. An overview off all themes and subthemes that emerged through the thematic analysis is presented in Table 4.

**TABLE 4:** Themes and Subthemes regarding the use of the Guardian system among older adults and their formal and informal caregivers.

Themes and subthemes
Theme: End-users experienced added value of the medication reminders and activity suggestions
Subtheme: Timing and context are important determinants of the relevance of reminders and suggestions
Subtheme: Although older adults indicated to always follow up on reminders and suggestions, they indicated to not perceive the SAR as persuasive
Theme: Participants expected to have more interaction with and through the Guardian system
Subtheme: Despite most participants expressing the limited interactivity as a shortcoming of the Guardian system, the older adults’ behaviour and comments indicated that they enjoyed having the SAR as company and were satisfied with the system
Theme: Some older adults felt ashamed of having a SAR in their home
Theme: Older adults perceived the SAR as a social actor
Subtheme: Human characteristics of the SAR are perceived as positive
Subtheme: Dutch participants perceived the physically embodied SAR as more robot-like than the virtual embodied SAR
Theme: Caregivers see potential for the Guardian system to support in reducing the care burden
Subtheme: The Guardian system can enhance communication between caregivers and older adults by providing conversation topics
Subtheme: Swiss caregivers indicated concerns that the SAR will replace human contact and take over their job as caregivers
Theme: Trust in the Guardian system and privacy concerns were experienced differently among different types of end-users
Subtheme: The sleep mode positively contributed to older adults’ experience of control over the SAR

### 3.1 Theme: end-users experienced added value of the medication reminders and activity suggestions

In the interviews that were conducted following the installations of the Guardian system, thus prior to the use of the system, most participants explained to not yet know what to expect from the Guardian system and whether it would be useful to them. Nevertheless, some older adults indicated they were impressed by the system and its various functionalities, and thought the system could be of use to them. After using the system for a while, most participants (i.e., older adults, formal caregivers and informal caregivers) indicated during the interviews that they perceived the system as useful, especially thanks to the medication reminders and activity suggestions. This notion was expressed among all three end-user groups during the interviews.

*“On a daily basis the system is nice, especially for medication reminders”* - Informal caregiver (NL)

Furthermore, some of the older adults expressed that they thought the system could even improve their autonomy. However, most of the participants also indicated they wanted to see improvements to the system. In addition, most older adults perceived the system as useful, but they indicated it would be especially beneficial for others who are more frail than themselves.

*“It [the Guardian system] could be useful for improving people’s autonomy, but it should work perfectly”* - Older adult (IT).

*“It’s not relevant yet because I don’t need any help yet. It could be more relevant for people who are a bit further in the dementia process”* - Older adult (NL).

*“It [the Guardian system] is important in the sense that it keeps me busy and stimulated, but I realise that perhaps it would be more useful for a person who is less independent than I am”* - Older adult (IT).

#### 3.1.1 Subtheme: timing and context are important determinants of the relevance of reminders and suggestions

While participants found the medication reminders and activity suggestions generally useful, interviews revealed that for certain situations some of the reminders or suggestions were not always perceived as relevant by the older adults. Some older adults explained that this was due to the mismatch between the system properties and the situation of use. For example, one of the older adults explained that the reminders were not suitable for her, as her medication requires a particular time of intake, depending on factors such as dinner times. However, the system was not able to deliver the reminders at these particular moments, due to too many contextual variables such as the exact time of finishing dinner.

*“The reminders do not always match the situation but I think it is nice to get a reminder. Especially the medication reminders”* - Older adult (NL)

#### 3.1.2 Subtheme: although older adults indicated to always follow up on reminders and suggestions, they indicated to not perceive the SAR as persuasive

The majority of older adults indicated that they did not find the SAR persuasive or influential in changing their daily routines. This was confirmed by the 7-point Likert-scale questions, where older adults disagreed with statements about the SAR’s influence. The statement ‘The SAR has an influence on me’ received average scores of 1.8 ± 1.2 from Swiss older adults, 2.1 ± 2.6 from Italian older adults, and 3.6 ± 2.3 (for Misty) and 2.5 ± 1.9 (for Lizz) from Dutch older adults. Similarly, the statement ‘The SAR makes me reconsider certain habits such as my diet, exercise pattern, or medication intake’, which was not measured among Swiss older adults, scored 1.6 ± 2.2 for Italian older adults and also low scores among Dutch older adults, with 2.5 ± 2.6 for Misty and 2.3 ± 2 for Lizz.

Despite these low scores indicating that the SAR is not very persuasive, older adults claimed to always adhere to the reminders and suggestions provided by the SAR. Thus, while the SAR’s perceived persuasiveness might have been low, older adults still followed its reminders and suggestions.

*“I do not immediately follow up on the reminders but in the end, I always did. But, it was always already in my head to do so, so I did not really need the reminders. Still, they are useful”* - Older adult (NL).

Similarly, most of the FCs and ICs were of opinion that the SAR did not influence the older adults. However, caregivers did notice positive reactions of the older adults towards the SAR. According to the caregivers, the older adults interacted with the SAR as if it was a real person. Therefore, most caregivers thought that the SAR could be helpful, especially with reminders on medicine intake and daytime structure. However, even though some of the caregivers explained that they saw the older adults feeling connected to the SAR, it was noted that this connection was not yet enough for the SAR to have a persuasive influence on the older adults.

As an example, during one of the interviews an IC elaborated on the potential effectiveness of the SAR for raising awareness about water intake for older adults. However, she doubted the robustness of the behaviour change after removing the system, thereby attributing a persuasive impact to the system. In contrast, the corresponding FC expressed scepticism about the system’s ability to bring about lasting changes in habits. Next to these varying perspectives, most other FCs were confident that the SAR was effective in reminding older adults about their appointments and medications, though they noted that the system’s persuasiveness was somewhat limited.

### 3.2 Theme: participants expected to have more interaction with and through the guardian system

According to the participants, the interaction with the Guardian system could still be improved with regard of the type of interactions that were facilitated, as well as the verbal interactions. Overall older adults indicated that they did not experience a strong sense of interaction with the SAR due to limited interaction possibilities, and their expectations regarding dialogue or reactions from the SAR were not met. Some older adults even felt frustrated when they tried to interact with the system, as the SAR did not work as they expected.

Almost all participants indicated that they believed the SAR lacked speech interactions. A common statement was, *“It’s a pity that it doesn’t respond to my voice”*. Although the older adults already liked the concept of the SAR as a companion, they indicated that they wanted to respond with speech to the spoken messages or commands and have more speech interaction with the SAR.

*“I would have liked to talk to the robot”* - Older adult (CH).

This lack of speech interactions was not in line with the expectations of the older adults. They expected a SAR with voice interactions, as they compared it to systems they already knew or used (e.g., Google Assistant or Siri). Therefore, some of the older adults were disappointed after the first few days of use.

*“The interaction is way too little. I expected more reactions to my answers. I would like to have interaction via speech”* - Older adult (NL).

Moreover, the designated way of interacting with the SAR, via a tablet, sometimes caused issues: some older adults in Switzerland faced difficulties when holding the tablet, as well as keeping it charged and connected to the internet.

*“Maybe it would have been better to be able to respond verbally instead of using the tablet, it all seemed complicated to me”* - Older adult (IT).

The FCs and ICs expressed similar opinions. Many caregivers believed that the SAR lacked adequate interaction and added that the tablet caused a barrier in the interaction between the SAR and older adults. Moreover, some of the caregivers added that the functionalities for interaction between older adults and caregivers could be made easier and more innovative by adding voice recognition and by the option to record alarming situations.

*“If the robot works like an Alexa system with voice recognition, the system will be more interesting and effective”* - Informal caregiver (CH).

*“There should be an emergency function, which translates what the elderly person says into a message”* - Formal caregiver (CH).

Besides the lack of speech interaction, older adults had more comments regarding interaction with the SAR. One expressed the desire to be able to respond with emoticons to messages sent by caregivers. Another older adult shared that the questions and responses from the SAR were always the same, not always relevant, and there was no feedback on the answers they provided.

*“There’s no real interaction with the robot. I keep on chatting to her, but she does not respond”* - Older adult (NL).

Similarly, the FCs and ICs noted the limited interactivity, particularly the lack of verbal responses to inquiries (i.e., limited feedback on the answers), and even indicated that the Guardian system is sometimes useless because of this. According to the FCs, it would be interesting if the interactivity of the SAR increased by, for example, suggesting relevant activities based on the responses of the older adults: instead of replying, *“it will be better tomorrow”*, the SAR could propose calling a relative, nurse, or doctor, whenever an older adult indicates to not feel well.

Subtheme: Despite most participants expressing the limited interactivity as a shortcoming of the Guardian system, the older adults’ behaviour and comments indicated that they enjoyed having the SAR as company and were satisfied with the system.

Even though all participants indicated they expected more interactivity of the Guardian system, most of the older adults liked interacting with the Guardian system. They perceived the SAR as a gadget, similar to a smartphone. Some described it as fun or enjoyable, particularly appreciating the sounds activated upon touch and the eye-contact that the SAR made, which were received positively.

*“It [the Guardian system] is a nice companionship. You know it is just a robot, but still, the companionship is nice”* - Older adult (NL).

Results from the 7-point Likert-scale questions showed that older adults were, on average, satisfied with the system. Except for Italian older adults (mean 2.5 ± 3.4), Dutch and Swiss older adults agreed with the statement “Overall, I am satisfied with the SAR” with scores ranging from 4.4 ± 1.7 to 6.2 ± 1.2. Additionally, the statement “I liked to use the SAR,” which was not answered by Swiss participants, received agreement from Dutch and Italian older adults with scores between 3.8 ± 4.1 and 7 ± 0.

Hence, the data from the interviews with older adults and their caregivers, supported by the Likert-scale questions asked to older adults during these interviews, indicate that older adults enjoyed having the SAR in their homes. This can be considered surprising given that all participants also expressed dissatisfaction with the limited interactivity of the Guardian system.

### 3.3 Theme: some older adults felt ashamed of having a SAR in their home

Some older adults indicated they felt ashamed of having a robot in their home. They felt that having such a robot could be a sign that they were in need for care, which some participants experienced as stigmatizing, as expressed during the installation sessions. One informal caregiver noted that Lizz was less noticeable due to its smaller size and less bulkiness, which could reduce the embarrassment associated with needing such help. However, some older adults perceived the small design of Lizz as a limitation, as Lizz could easily be pushed over during use and therefore, they preferred Misty.

*“Lizz seems better because it’s less notable, so probably less embarrassment is experienced”* - Formal caregiver (NL)

### 3.4 Theme: older adults perceived the SAR as a social actor

Some older adults believed that the SAR could be an effective social companion. They mentioned that the SAR could help alleviate loneliness and even be considered as *“a member of the family”*. Some of the older adults shared that they expected to miss the SAR once it was de-installed. Other older adults were more sceptical about the SAR’s ability to improve social aspects like loneliness. Nevertheless, they felt that future enhancements of the system could better support connections, not only between themselves and the SAR but also with caregivers. They emphasized the need for more response options to messages from caregivers and questioned whether FCs actively engaged with the system. They particularly appreciated ICs using the application.

Finally, FCs and ICs considered the SAR as good companionship as the older adults gradually became accustomed to its presence. One of the Swiss formal caregivers said:

*“It is clear that the robot can reduce the loneliness of its users because over time you get used to having Misty at home and you are not alone anymore”* - Formal caregiver (CH).

Although most FCs were positive about the use of the SAR as a social companion, 2 FCs shared their fear of the SAR replacing human contact and leading to a loss of social ties.

#### 3.4.1 Subtheme: human characteristics of the SAR are perceived as positive

During the interviews, older adults indicated that the appearance of Misty was *nice, sweet, friendly* and *cute*. Participants responded positively to the appearance of the SAR, and perceived the SAR as a character with human-like attributes. Some particularly liked Misty’s eyes. The older adults gave the SAR names and were engaged in their conversations with the SAR. However, a couple of older adults was negative about the SAR, e.g., the size of the SAR or its noise caused by constantly charging it. One older adult initially felt apprehensive about the robot’s large eyes, especially during moments when his mood was lower and he wanted to avoid eye contact with the SAR. However, over time, he became positive about Misty, indicating that he just needed to get used to it. Similar to the responses of most of the older adults, almost all of the FCs and ICs were positive about the design of Misty.

*“Friendly appearance of the robot: eyes, mimics, sound when touched”* - Informal caregiver (CH).

#### 3.4.2 Subtheme: dutch participants perceived the physically embodied SAR as more robot-like than the virtual embodied SAR

Participants experienced Misty and Lizz as similar in concept and functionality, noting no significant differences in their messages, reminders or suggestions.

*“No, there is not really a difference. Except for the appearance and the layout, they were very much alike.”* - Formal caregiver (NL).

Misty’s larger size and noise were sometimes noted as drawbacks, while Lizz’s smaller size made it more suitable for small living spaces. Overall, participants found both robots useful but desired more interactivity, such as the ability to have conversations with them. In terms of interactivity, participants appreciated Misty’s sounds and idle movements that were activated upon touch. Preferences for Misty or Lizz seemed based on personal taste, with some participants favoring Lizz’s cute appearance and others preferring Misty’s more robotic look.

*“That other robot (Misty) is more really a robot”* - Older adult (NL).

*“Lizz is just a tablet and is less capable of interaction. Misty feels more like a human being in the house and therefore perceived as more ‘present”* - Older adult (NL).

### 3.5 Theme: caregivers see potential for the Guardian system to support in reducing the care burden

Caregivers viewed the Guardian system as supportive in their work. Some caregivers also tried to incorporate the caregiver application into their daily tasks by regularly consulting it. They indicated that the system helped them to stay informed about the older adults’ wellbeing and thought the system could also provide peace of mind for informal caregivers by allowing them to monitor their relatives.

Caregivers saw potential in the Guardian system to improve their work efficiency, for instance, by reducing unnecessary client check-ups, prioritizing clients who need care, or engaging in more informed conversations about clients’ wellbeing. However, they felt that the Guardian system still required further development, as it was not yet sufficiently advanced. Some FCs noted that the system would not replace their tasks entirely since they still needed to verify if reminders were followed.

Although the interviews showed that the Guardian system could still be improved, some caregivers indicated that the current system could already stimulate older adults to be more active. Additionally, some FCs expressed that the system resulted in them not needing to call or bother older adults daily, a change that reduced their care burden, which they appreciated. Additionally, some FCs felt that the older adults’ health-related self-reports could support early detection of health issues, potentially making care more efficient.

#### 3.5.1 Subtheme: the Guardian system can enhance communication between caregivers and older adults by providing conversation topics

Some of the caregivers indicated that the SAR not only helped relieve their care burden but also provided conversation topics during visits with older adults. The SAR itself sparked discussions, and the entire Guardian system offered insights into clients’ health-related issues by the responses to the self-reports. These self-reports led to conversation topics as well.

*“It resulted in more conversation topics which was a positive experience”* - Formal caregiver (NL).

#### 3.5.2 Subtheme: Swiss caregivers indicated to be concerned that the SAR will replace human contact an take over their job as caregivers

Some caregivers in Switzerland shared concerns that a SAR might not only take over certain job responsibilities but could also reduce human interaction for older adults. Two FCs shared their fear of the SAR replacing human contact and leading to a loss of social ties. Hence, they were doubting whether the SAR could reduce loneliness, saying:

*“I am not sure a robot can actually make you feel less lonely”* - Formal caregiver (CH)

Other FCs and ICs noted that Misty offers pleasant companionship and has become part of a daily routine for the older adults.

*“It is clear that the robot can reduce the loneliness of its users because over time you get used to having Misty at home and you are not alone anymore”* - Formal caregiver (CH).

### 3.6 Theme: trust in the Guardian system and privacy concerns were experienced differently among different types of end-users

Trust in the Guardian system varied among different end-user groups. The majority of older adults felt comfortable with the system, largely because they trusted their ICs and FCs who were involved in its setup and use. This trust in their caregivers seemed to help them accept the system’s presence in their homes, with many older adults expressing little to no concerns about their privacy. Some older adults mentioned they had *“nothing to hide”*, and one person indicated that he felt that privacy is already limited in today’s world, making the privacy trade-offs associated with the SAR feel acceptable, as they felt privacy is largely nonexistent anyway.

*“I have no problems with the privacy. Our data is everywhere, if we want to be safe, we should not even use our cell phones. Nowadays, our data is everywhere, so if we choose to use these technologies, we just have to know about it and accept it, so no problem for me”* - Older adult (IT).

However, ICs and FCs were more cautious, with regard to their trust in the Guardian system and they also shared some privacy concerns about the data protection and the usage of the camera and microphone. This difference between older adults and their caregivers reflects the caregivers’ stronger emphasis on data security, which they saw as essential, particularly when caring for vulnerable individuals like older adults.

*“I have no problem with it [privacy] in my own situation. I do already have a smart speaker. If you want to interact, you have to be flexible with this”* - Older adult (NL)

*“Depends who has access to the data, especially the videos. If it remains within our organization it is okay. We had informal caregivers who installed webcams in the home of their loved one to monitor what is going on. This means they can also see what I’m doing”* - Formal caregiver (NL)

*“You must be able to cover the camera with for example a thing like you put on your webcam”* - Formal caregiver (NL)

#### 3.6.1 Subtheme: the sleep mode positively contributed to older adults experience of control over the SAR

Older adults generally felt a strong sense of control and trust in the Guardian system. Key features that contributed to this feeling included the ability to mute the system, its ease of use, and the way the SAR reliably performed its programmed tasks. One older adult highlighted this sense of control, saying:

“Yes absolutely [I have the feeling of control], I am the one who decides when and what to report to the robot” - Older adult (IT).

However, while the system contributed to feelings of autonomy and control, some older adults indicated that they felt not in control since they were not able to fully operate the system (i.e., usability issues with operating the tablet or not being able to set appointments in the system by themselves). Furthermore, some older adults indicated that the system did not influence their perception of safety, indicating they felt neither more secure nor at risk due to its presence.

“I don’t feel more safe, but I don’t feel in danger either. I don’t think it [the Guardian system] can have an influence on this dimension” - Older adult (IT).

## 4 Discussion

Socially Assistive Robots (SARs) have the potential to enable older adults to age at home and support the defragmentation of care (Ienca et al., 2017; Robben et al., 2012). However, their adoption and acceptance rates are relatively low (Greenhalgh et al., 2017; Getson and Nejat, 2022). One possible reason is that SARs might not fully address all user needs. Therefore we presented a case study in which a SAR was iteratively developed and user experience was evaluated in a real-life context involving all three primary end-users (i.e., older adults and their formal and informal caregivers). The aim of this case study was to identify factors that can influence user experience and to translate those insights into design considerations for SARs, in order to better adjust SARs to the user needs of older adults. In the first usability study related to this project (Ciuffreda et al., 2023a), important user requirements for the Guardian system were identified as monitoring, reminding, and social companionship. Progress through iterative development in the project and results from alpha testing demonstrated the system’s potential to address these needs (Amabili et al., 2023). However, it also highlighted a desire for more interaction and personalization, particularly in terms of the robot’s appearance, including embodiment, and communication methods. In addition, ethical concerns were raised, such as the inability for older adults to mute the system by themselves. Therefore, these components were added to the guardian system and evaluated in the current study, and hence, we focused on personalisation, interactivity, embodiment, and ethical considerations, while also exploring additional factors that may impact older adults their user experience of the SAR.

Results suggest the relevance of using a SAR in care for older adults, especially for medication reminders and activity suggestions. By adding more personalisation options, the system is expected to be even more useful, as became clear from the interviews. Formal and informal caregivers indicated that additional functions were preferred and that the system should be tailored to user preferences and context, as this would enhance the expected usefulness. Such a relation between personalisation and expected usefulness was also found in earlier research (Ko et al., 2009). Additionally, in the Netherlands, where the SAR was more extensively personalised than in Italy and Switzerland (i.e., Dutch researchers did personalise message content and reminders to personal details of older adults), personalised messages were well-received. Older adults in the Netherlands appeared more enthusiastic about the messages and reminders, likely due to the higher level of personalisation. In explanation, during the interviews, many Dutch participants quoted the SAR’s messages with expressions of appreciation, with some noting that the personalised reminders were useful. In contrast, older adults in Italy and Switzerland did not recall the reminders or messages. Therefore, these results suggest that personalisation may enhance the user experience with SARs, supporting findings from earlier research by Gasteiger et al. (2021), Hofstede et al. (2022), Shryock and Meeks (2022) and Di Nuovo et al. (2018), which found positive effects of personalisation on acceptance and perceived usefulness of SARs. Moreover, Sebri and Savioni (2020) noted that a lack of personalisation in gerontechnologies often results in these technologies being seen as useless. This aligns with our findings, as medication reminders were sometimes perceived as useless due to their limited adaptability to individual contexts and medication schemes.

Furthermore, the results of the field study suggest that low interactivity could result in user frustration and unmet expectations. Therefore, it seems that interactivity is an important factor to create a positive user experience. This finding resonates with the findings of Getson and Nejat (2021), who determine limited interaction possibilities as an important barrier to positive user experiences with SARs in healthcare. During the interviews, participants particularly expressed a desire for more speech interaction, some even identified its absence as a barrier to ease of use and system satisfaction, a finding also supported by the research of Getson and Nejat (2021). The SAR’s reliance on a tablet for interaction further emphasized these challenges, as not all participants felt comfortable using such devices. Many expressed a clear preference for voice-based interaction, noting the system’s interaction richness as insufficient, an issue also identified by Pednekar et al. (2023). These findings resonate with research by Lima et al. (2021), which underscores the potential of enhanced interactivity to improve user experiences with SARs. Additionally, Gao et al. (2010) highlight the importance of interaction richness in promoting sociability and overall user satisfaction. Increasing voice-based interactivity could therefore significantly enhance the ease of use and acceptance of SARs among older adults.

Despite limited interactivity, most participants appreciated the SAR as a companion, with some perceiving it as a social actor they would miss after de-installation. This suggests the potential of the SAR to address loneliness while supporting daytime structure through reminders and activity suggestions (Broekens et al., 2009; Moro et al., 2019). Furthermore, most of the caregivers perceived the SAR as useful to foster social interaction between them and their clients or relatives, by means of monitoring and discussing the monitored activities and wellbeing reports of the older adults. However, most of the caregivers expressed a preference for calling older adults and maintaining more direct contact. This aligns with findings from Czaja et al. (2013), which indicate more feasibility of interventions based on direct interactions compared to indirect interactions. Similarly, IJsselsteijn et al. (2020) identified a mismatch in many assistive technologies, where offered monitoring solutions often failed to meet the desire for warmer, more personal interactions. Our findings resonate with these insights: while monitoring capabilities of assistive technologies can reduce caregiver burden and facilitate more conversational topics, both older adults and caregivers indicated to value warm, direct contact. Hence, balancing these two aspects seems essential, as they often appear to compete with one another. To address this, Ahmed et al. (2016) advocate for multi-modal solutions, allowing for diverse communication methods that cater to varying needs. Such an approach could enhance the social connectedness of SARs by enabling users to choose the communication mode best suited to a given situation. This flexibility could improve the system’s ability to meet the emotional and practical needs of its users.

The interviews revealed that older adults especially valued the active engagement of their informal caregivers through the SAR. Thus, next to the engagement of older adults and their formal caregivers with the system, informal caregivers also actively used it. This feedback supports the idea that SARs can help engage multiple groups of caregivers (i.e., formal and informal caregivers), and thence, reduce fragmented care by fostering communication across different types of users (Getson and Nejat, 2021). Moreover, SARs can also stimulate older adults to communicate more, enabling better information sharing with caregivers, which can help reduce fragmented care by providing a clearer understanding of their needs and health status. Studies by Wada et al. (2005) and Moyle et al. (2014) have shown that even low-complex robots, like the robotic pet Paro, enhance social interaction and encourage older adults to communicate more. Similarly, SARs can be intentionally designed to promote communication between users (De Graaf et al., 2015), and this was reflected in our research. However, the older adults in our study also suggested features to enhance caregiver interaction, implying contact with their caregivers as a user need, but also implying that they were not yet fully satisfied with the current communication capabilities between themselves and their caregivers. Although some participants questioned whether all formal caregivers were fully utilizing the SAR, they valued the communication it enabled with their informal caregivers and expressed a desire for more response options to further increase contact.

Participants appreciated the human-like characteristics of the SAR, and some indicated that these traits even enhanced their perception of the robot as a social actor. To some extent, this observation aligns with the uncanny valley effect described by Seyama and Nagayama (2007), which suggests that affinity increases as robots appear more human-like, but beyond a certain point, if they become too human-like, robots can evoke unease and drastically reduce affinity. Some participants found the physical embodiment of Misty to be more advanced compared to Lizz. For others, the virtual character of Lizz was more appealing. Physical embodiment contributing to affinity can be explained in two ways: on the one hand, physical embodiment may lead to a greater sense of affinity, as it can be perceived as more human-like to have physical embodiment. On the other hand, it can also decrease affinity, as some participants indicated that the physical embodiment made Misty seem more robot-like, and hence, less human-like. This suggests a complex interplay between physical appearance and emotional connection, where a SAR’s level of embodiment may both enhance or hinder user experience depending on personal preferences. As was found by Pino et al. (2015), a mismatch between user needs and aspects such as the appearance of a SAR is one of the most important barriers to SAR adoption. Therefore, taking into account the embodiment of the SAR, and aligning it to user preferences, seems to be a relevant consideration in the design of a SAR.

In terms of discussing ethical considerations, the interviews made clear that trust and privacy concerns varied across user groups. Compared to caregivers, older adults expressed fewer concerns about ethical risks and seemed to experience challenges in identifying such risks. Fiske et al. (2019) noted that participants often experience it challenging to identify concerns related to trust and privacy, hence, a finding reinforced by our interviews with older adults. Still, caregivers offered valuable insights into the design process to reduce ethical risks. For example, the inclusion of a sleep mode was positively received by older adults as they felt more in control. Participants also expressed dissatisfaction with the inability of older adults to set appointments and reminders themselves. This limitation, noted by both older adults and caregivers, reflects findings from Liu and Liao (2021), which suggest that restricted autonomy can reduce perceived ease of use. Therefore, ethical considerations such as fostering autonomy, providing control, and addressing trust and privacy concerns are crucial in SAR design. Furthermore, our findings not only imply the importance of addressing ethical issues (Liu and Liao, 2021; Lukkien et al., 2023), but also highlight the value of involving multiple primary end-user groups as they can complement each other and offer diverse perspectives. According to Suijkerbuijk et al. (2019), actively involving end-users in the design process is particularly important when developing technology for people with dementia. Therefore, this seems relevant to our study as well, since our participant sample also included individuals with dementia. Moreover, Suijkerbuijk et al. emphasize the value of involving people with dementia not only in evaluative phases but also as co-designers in earlier design stages.

In our field study, some older adults reported feeling stigmatized by having a SAR in their homes, as it symbolized their need for care. Such emotional responses seem to emerge only after prolonged use in one’s own environment. This observation aligns with McGlynn et al. (2014) and underscores the importance of conducting long-term field studies. Similarly, some participants required time, sometimes up to a week, to acclimatize to features such as the robot’s large eyes, after which they began engaging more actively. Such adaptation would be difficult to observe in the short durations typical of lab research (Smarr et al., 2012). Additionally, the challenges of maintaining system stability in real-life environments, where variables are less controlled, provided valuable insights into the robustness and usability of the SAR, building on findings by Papadopoulos et al. (2020). Moreover, some participants expressed dissatisfaction when the SAR was uninstalled after the study, indicating that they had integrated it into their daily routines. This reaction highlights the emotional and practical connections participants can develop with assistive technologies during field studies, providing insights critical for designing systems that truly meet user needs. In conclusion, field studies enhance the external validity of findings by uncovering important factors such as emotional responses, adaptation time, and system stability, which might remain hidden in controlled environments.

### 4.1 Limitations

To balance participant burden with the unique research opportunities available, our study adopted flexible protocols across countries. For example, we conducted a longer testing period in Italy compared to Switzerland and the Netherlands, tested two different types of SARs in the Netherlands, and included more personalisation options in the Netherlands than in Italy and Switzerland. These variations allowed us to expand our research scope and address unique aspects of SAR use in different contexts. In Switzerland, recruitment challenges led us to include three focus groups, limiting participant burden and prioritizing ethical considerations, an approach resulting from the increased ethical awareness fostered by Responsible Innovation (RI) workshops among project members. Furthermore, the challenges in collecting quantitative data, particularly with older adults, were an important aspect of our study. Not all participants fully understood the questions, and the process was time-consuming, leading to incomplete data for some individuals. In response, we adapted a flexible data collection protocol that varied by country, allowing us to adjust to the specific needs of our user groups. As a result, we agreed to collect less quantitative data and focus more on the qualitative data, which was in some situations more appropriate to collect successfully. This decision reflects our commitment to obtaining the most reliable and meaningful data for our study aligned with RI principles. Given the incomplete nature of the quantitative results, we decided it was most appropriate to place them in the Supplementary Material. This approach ensures that the main findings of the study remain focused on the qualitative data, while still providing transparency by including the quantitative results for reference. We prioritised the qualitative aspects of the study, which provided deeper insights, and we made sure to reference the quantitative data where relevant, ensuring a balanced presentation of the findings. While the tailored protocols provided flexibility and enabled unique research opportunities, they also underscore an important consideration in study design: the balance between standardization and adaptability. When the goal is to enable direct cross-country comparisons, more aligned protocols are necessary to minimize confounding factors and enhance the robustness of the results. In contrast, an adaptable design can be highly effective for gathering diverse insights and seizing unique research opportunities, such as extended testing periods or additional customization options. However, this approach may limit the reliability of cross-country comparisons. This balance is further influenced by principles of RI. Ensuring ethical research practices and minimizing participant burden often necessitates tailoring protocols to local contexts, even if it leads to deviations from a standardized approach. These adjustments prioritize participant wellbeing and respect for local needs but may introduce variability that complicates cross-country analyses. Ultimately, the choice between standardization and flexibility depends on the research objectives, requiring careful consideration of the trade-offs between robust comparisons, contextual insights, and ethical integrity.

The findings reveal a limitation in the study regarding the selection and responses of the user group. It is unclear whether the older adults included were the most suitable participants to evaluate the SAR, as many perceived it as more appropriate for individuals with greater needs. This raises the question of whether the system was misaligned with their actual requirements or whether these participants were not representative of the intended end users. An alternative explanation is that the older adults may have overestimated their own capabilities, as suggested by prior research (Hirsch et al., 2000; Sakurai et al., 2013). This cognitive bias is supported by discrepancies in the data: while participants reported engaging with the system (e.g., listening to reminders and following its advice), they rated its persuasiveness and relevance poorly. This explanation is further reinforced by caregiver feedback, as caregivers observed older adults engaging with the system and identified features like medication reminders as valuable—findings consistent with Vercelli et al. (2018). This suggests that caregivers may provide a more accurate assessment of older adults’ actual needs and system usability, in line with research by Miller et al. (2013). However, a limitation of this study is its inability to clearly distinguish between these two interpretations: whether the issue lies with the user group selection or with the self-perception biases of the older adults. Addressing this ambiguity would require more targeted methods to account for such biases or broader triad testing involving caregivers alongside older adults to capture a more holistic understanding of user needs and system effectiveness. Furthermore, collecting data on the number of interactions is also an important recommendation for future research since it can lead to deeper insights in the understanding of user engagement and the analysis of usage patterns compared to the interview data.

Finally, although the SAR and its applications were generally seen as easy to use, some participants raised concerns about system stability. Malfunctions led to unmet expectations, which, according to Matarić (2017), can negatively impact user experience. For some older adults, the system’s instability caused frustration, potentially affecting the study results (Amsel, 1992), which can be considered a limitation of the case study. However, involving users at every stage of the design process is crucial to better align the system with user needs (Steen et al., 2007; Suijkerbuijk et al., 2019). Despite the low system stability of early prototypes, user feedback remains valuable for refining the design in every design phase (Lim et al., 2006). Therefore, we acknowledge this as a limitation but accept it as an inherent challenge when aiming to involve users in every phase of the design process.

### 4.2 Recommendations for SAR design

#### 4.2.1 Personalisation: include personalized communication and adapt reminders to user context

Personalisation emerged as an important factor in the acceptance and perceived usefulness of SARs. Tailoring reminders, activity suggestions, and communication options to individual preferences and contexts is expected to enhance their relevance and engagement with the SAR. Research indicates that personalised systems are more likely to meet user needs and improve usability (Gasteiger et al., 2021; Hofstede et al., 2022), findings that can be supported by our study. Therefore, we recommend incorporating personalisation options, such as the ability to customize communication styles and messages, appearance-related variables, and medication schedules, and providing multi-modal interaction methods, while considering the varying preferences and needs of users in different contexts.

#### 4.2.2 Interactivity: increase interaction richness and include speech

Based on the interview data, an interactive SAR seem important to create intuitive and satisfying user experiences. Older adults often find speech interaction more accessible and natural than tablet-based interfaces Pednekar et al. (2023). Despite positive feedback on interactive features, such as touch-responsive sounds and vocables, the absence of robust speech interaction was frequently identified as a barrier to ease of use and system satisfaction in our study. Studies suggest that rich and flexible interaction options, particularly those including speech, can significantly improve perceived sociability and user engagement Gao et al. (2010). Prioritizing multimodal interaction methods, including speech, touch, and gestures, aligns with findings by Lima et al. (2021), which emphasize the importance of designing interaction mechanisms that accommodate diverse user abilities.

#### 4.2.3 Embodiment: align physical or virtual embodiment with user preferences

The physical embodiment of SARs plays a role in shaping user perceptions. While some users find physically embodied robots more engaging and human-like, others may perceive them as more robot-like. Virtual characters may appeal to some users due to their non-intrusive nature, whereas others may prefer physical robots for their sense of presence and advancement. Addressing this variability requires offering flexible design options and aligning embodiment with user preferences, as mismatches in appearance and functionality can act as barriers to adoption Pino et al. (2015).

#### 4.2.4 Ethical considerations: ensure autonomy and user control

Designing the SAR such that it empowers users to exercise autonomy and experience control seemed an important ethical consideration. Older adults expressed dissatisfaction when they were unable to set their own reminders or customize system features, a limitation that aligns with findings by Liu and Liao (2021), which link restricted autonomy to reduced ease of use and satisfaction. We found that features like a sleep mode can support autonomy, even while such trust and privacy concerns were sometimes expressed by other end users because older adults seemed to experience challenges in expressing ethical concerns (Fiske et al., 2019).

#### 4.2.5 Connectedness: balance monitoring and warm contact

Our study revealed the importance of balancing technological monitoring capabilities with the desire for warm and personal interaction as an important challenge in SAR design. While caregivers appreciated the system’s ability to track activities and generate conversational topics, they also stressed the value of direct, human contact. Older adults similarly valued reminders and activity suggestions but expressed a preference for communication that felt personal and empathetic. Research by IJsselsteijn et al. (2020) points to the need for warmer assistive technologies that avoid the depersonalization often associated with monitoring tools. Incorporating multimodal communication features, as advocated by Ahmed et al. (2016), could offer flexibility, allowing users to alternate between monitoring and direct contact based on their emotional and practical needs. Currently, one communication mode was used, creating an imbalance. While multiple options won’t guarantee balance, users have expressed a desire for both monitoring and direct contact. It can be expected that this flexibility will allow them to choose the approach that best suits their needs, helping them find the right balance for themselves.

#### 4.2.6 Dignity: minimize the stigmatizing effects of assistive technologies for older adults

Participants in our field study occasionally reported feeling embarrassed about having a robot in their home, particularly if they experienced it as highlighting their perceived dependency. This aligns with findings by McGlynn et al. (2014) and IJsselsteijn et al. (2020), which emphasize the need for designs that do not inadvertently reinforce stereotypes about ageing or frailty. Minimizing stigma involves careful attention to appearance and functionality. Additionally, involving users early in the design process can ensure that the final product aligns with their expectations and preferences, reducing feelings of discomfort or stigma (Smarr et al., 2012).

### 4.3 Conclusion

Our study identified six factors to consider in the design of SARs for older adults: personalisation, interactivity, embodiment, ethical considerations, connectedness, and dignity. Based on the interview data, personalisation and interactivity emerged as particularly important among these factors for potentially fostering positive user experiences for older adults and their caregivers. While these two factors appeared to be prioritized by participants, the study also highlighted autonomy and control as significant ethical considerations. Additionally, addressing user preferences related to embodiment, minimizing stigma to uphold dignity, and taking connectedness into account by balancing monitoring with warm contact, were also suggested as factors that could enhance SARs’ ability to meet the emotional, functional, and social needs of older adults. Moreover, iterative co-design and real-world implementation were found to be valuable, as it may expose issues such as system stability or the impact of stigma, both of which can affect user experience, and it may confirm iterative design choices such as the autonomy increase by the mute function. Finally, our study suggests that involving complete triads (older adults and their formal and informal caregivers) in the testing process can provide a more holistic understanding of user needs and ensure more comprehensive attention to ethical concerns, particularly as users may face challenges in articulating such concerns independently.

## Data Availability

The raw data supporting the conclusions of this article will be made available by the authors, without undue reservation.
