# Supplementary material for: A field study to explore user experiences with socially assistive robots for older adults: emphasizing the need for more interactivity and personalisation
Source: Front Robot AI. 2025 Mar 28;12:1537272. doi: 10.3389/frobt.2025.1537272 (PMC12015597; doi:10.3389/frobt.2025.1537272)
Supplement: Supplementary file 1 [file Supplementaryfile1.pdf]

Supplementary Material

1 ANSWERS TO LIKERT-SCALE QUESTIONS BY OLDER ADULTS

Table S1. Quantitative data of older adults

Statement	Switzerland		Italy		The Netherlands			
	Mean	SD	Mean	SD	Misty: Mean	SD	Lizz: Mean	SD
**Usability survey**								
It was easy to use the SAR	5.1	2.4	4.1	4.8	6.7	.75	6.9	.3
I feel comfortable while using the SAR	4.9	2.6	4.3	4.7	7	0	6.3	1.4
It was easy to learn how to use the SAR	.	.	4.8	5	6.3	1.5	7	0
The given information about the SAR was easy to understand	5.3	2	5	6	7	0	6.5	1
The interface of the SAR was pleasant	4.4	2.4	5.9	6.4	6.3	.94	5.7	1.9
I liked to use the SAR	.	.	3.8	4.1	7	0	6.3	1.3
**Perceived Persuasiveness Questionnaire**								
The SAR has an influence on me	1.8	1.2	2.1	2.6	3.6	2.3	2.5	1.9
The SAR is personally relevant to me	3.1	2.4	2	2.6	2.2	2.4	2.9	2.2
The SAR makes me reconsider certain habits such as my diet, exercise pattern or medication intake	.	.	1.6	2.2	2.5	2.6	2.3	2
**IMPACT survey**								
The SAR has all the features and capabilities I expected	2.5	2	3.3	3.7	3.8	2.3	3.1	1.8
**IBM Questionnaire**								
Overall, I am satisfied with the SAR	4.3	2.4	2.5	3.4	6.2	1.2	4.4	1.7

2 INTERVIEW SURVEY

Below, the interview survey that was used for older adults is shown. Please note that the researchers at the three different pilot sites used translated versions of the interview survey to ask the participants in their preferred language.

1 Senior

1.1 Information & recruitment session

<table>
<tr><th colspan="6">Demographics</th></tr>
<tr><td>Gender</td><td colspan="2">☐ Male</td><td colspan="2">☐ Female</td><td>☐ Other</td></tr>
<tr><td>Country senior lives:</td><td colspan="5"></td></tr>
<tr><td>Education level</td><td colspan="2">☐ Lower</td><td colspan="2">☐ Medium</td><td>☐ Higher</td></tr>
<tr><td>Living Situation</td><td colspan="2">☐ Alone</td><td colspan="2">☐ Living together with partner</td><td>☐ Other, namely:</td></tr>
<tr><td rowspan="2">Technology tools</td><td colspan="5">Which of the following devices do you often (more than once a week) use?
I'm using a… (multiple answers possible)</td></tr>
<tr><td colspan="2">☐ Computer</td><td colspan="2">☐ Tablet</td><td>☐ Smartphone</td></tr>
<tr><td rowspan="2">Technology level</td><td colspan="5">How do you rate your experience with technology (e.g. video calling, e-mailing, using whatsapp) on a scale of 1 (no experience) till 5 (much experience)?</td></tr>
<tr><td>1. None
☐</td><td>2. A bit
☐</td><td>3. Moderate
☐</td><td>4. Quite some
☐</td><td>5. Much experience
☐</td></tr>
<tr><td>Level of professional care</td><td colspan="5">How many hours of home care do you receive per week ?

What is their role? What do they do?</td></tr>
<tr><td>Level of informal care</td><td colspan="5">Do you have other people that support you?

What is their role ? What do they do?</td></tr>
</table>

1.2 Training & Equipment session at senior's home

**Functionalities & first impression**	
Which functionalities will be tested by the senior?	☐ Medication<br>☐ Meal<br>☐ Wellbeing<br>☐ Sleep Quality<br>☐ Activity Suggestions
What is the reason for this choice of functionalities?	
Ask after introduction and demonstration of the Guardian system	
What is your first impression of the Guardian System?	
Is everything clear? What did you think of this information session?	
Schedule debriefing session	

1.3 Follow-up after 1 week (15 min)

First impression & usability	
What is your impression of the Guardian system after using it for a week?	
How often did you use Guardian and what for? How did it go?	
Is Guardian easy to use on a daily basis? If so, why?	
Did you encounter any problems? If so, what problems and what did you do?	
Did you need support to use the system? If so, who helped you and how? Could you do it now by yourself?	

Did you enjoy using Guardian?	
How do you perceive the interaction with the robot?	
Did you talk to the robot?<br>If so, did you like it or not?	
Do you have any other comments?	

1.4 Debriefing session - Interview

Qualitative questions: usability, willingness to pay, impact, ethics	
Give a short summary of what the participant said during the follow-up: *We spoke to each other last week and you said …*<br>*Was something different last week? How did it go then?*	
Was there a moment you turned off the system yourself? If so, why?	
Do you feel confident while using the Guardian system?	
Do you believe Guardian as you used it the last two weeks is useful in your daily life? Why?	
Which of the functionalities you tested did you find valuable/useful? And which not? Why? Do you miss anything?	

You have used Guardian for 2 weeks now, how was the interaction between you and your informal and formal carer?<br><br>*Has anything changed?*<br>*How did you experience that?*<br>*Positive or negative?*	
Did you find the system sufficiently personalized?<br>If so, why? If not, what would you add?	
How do you feel about your privacy when using the robot?<br>Do you feel that your privacy is sufficiently respected? Or how can this be improved?	
Do you have the feeling of being in control over the actions taken by the robot in your home?	
To what extent do you trust the robot? Are there any things that you do not want or like the robot to do?	

<table>
<tr><td>Do you feel safer (and better watched over) when using the robot?</td><td colspan="20"></td></tr>
<tr><td>Do you see any risks or negative effects of using the robot, which we did not discuss?</td><td colspan="20"></td></tr>
<tr><td rowspan="2">What are you willing to pay for such a system as GUARDIAN? If the participant is not willing to pay: What’s stopping you from paying?</td><td colspan="5">☐0,- € per month</td><td colspan="5">☐5,- € per month</td><td colspan="5">☐10,- € per month</td><td colspan="5">☐15,- € per month</td></tr>
<tr><td colspan="20">or I’m willing to pay __________ ,- € once</td></tr>
<tr><td>Read the statements to the participant and ask them to indicate how strongly they agree or disagree on a scale of 1 – 5.</td><td colspan="4">1. Strongly Disagree</td><td colspan="4">2. Disagree</td><td colspan="4">3. Neutral</td><td colspan="4">4. Agree</td><td colspan="4">5. Strongly Agree</td></tr>
<tr><td>Using Guardian helps me to take my medication on time</td><td colspan="4">☐</td><td colspan="4">☐</td><td colspan="4">☐</td><td colspan="4">☐</td><td colspan="4">☐</td></tr>
<tr><td>Using Guardian helps me to become more active</td><td colspan="4">☐</td><td colspan="4">☐</td><td colspan="4">☐</td><td colspan="4">☐</td><td colspan="4">☐</td></tr>
<tr><td>Using Guardian helps me to eat and drink enough</td><td colspan="4">☐</td><td colspan="4">☐</td><td colspan="4">☐</td><td colspan="4">☐</td><td colspan="4">☐</td></tr>
<tr><td>Guardian system helps me to make me feel less lonely</td><td colspan="4">☐</td><td colspan="4">☐</td><td colspan="4">☐</td><td colspan="4">☐</td><td colspan="4">☐</td></tr>
<tr><td>Using GUARDIAN helps me to feel more independent</td><td colspan="4">☐</td><td colspan="4">☐</td><td colspan="4">☐</td><td colspan="4">☐</td><td colspan="4">☐</td></tr>
<tr><td>GUARDIAN helps me to have a daily routine</td><td colspan="4"></td><td colspan="4"></td><td colspan="4"></td><td colspan="4"></td><td colspan="4"></td></tr>
<tr><td>GUARDIAN helps me to inform my caregivers about my wellbeing</td><td colspan="4"></td><td colspan="4"></td><td colspan="4"></td><td colspan="4"></td><td colspan="4"></td></tr>
<tr><td></td><td colspan="4"></td><td colspan="4"></td><td colspan="4"></td><td colspan="4"></td><td colspan="4"></td></tr>
</table>

Where your experiences using GUARDIAN in line with your expectations prior to the test? Why?	

1.5 Debriefing session - Questionnaires

Think about all the tasks that you have done with the GUARDIAN system while you answer the following questions. Please read each statement and indicate how strongly you agree or disagree with the statement by circling a number on the scale. If a statement does not apply to you, circle N/A.

Note for researcher: You are allowed to use the opposite scale as well if that works better in your country but please make clear which scale is used.

IBM Usability Questionnaire								
	**Strongly agree**						**Strongly disagree**	
1. Overall, I am satisfied with how easy it is to use the GUARDIAN system.	1 ☐	2 ☐	3 ☐	4 ☐	5 ☐	6 ☐	7 ☐	N/a ☐
2. It was simple to use the GUARDIAN system.	1 ☐	2 ☐	3 ☐	4 ☐	5 ☐	6 ☐	7 ☐	N/a ☐
3. I could (effectively) successfully complete the tasks and scenarios using the GUARDIAN system.	1 ☐	2 ☐	3 ☐	4 ☐	5 ☐	6 ☐	7 ☐	N/a ☐
4. I was able to complete the tasks and scenarios quickly using the GUARDIAN system.	1 ☐	2 ☐	3 ☐	4 ☐	5 ☐	6 ☐	7 ☐	N/a ☐
5. I was able to efficiently (quickly) complete the tasks and scenarios using the GUARDIAN system.	1 ☐	2 ☐	3 ☐	4 ☐	5 ☐	6 ☐	7 ☐	N/a ☐
6. I feel comfortable using the GUARDIAN system.	1 ☐	2 ☐	3 ☐	4 ☐	5 ☐	6 ☐	7 ☐	N/a ☐
7. It was easy to learn to use the GUARDIAN system.	1 ☐	2 ☐	3 ☐	4 ☐	5 ☐	6 ☐	7 ☐	N/a ☐
8. I believe I could become productive quickly using the GUARDIAN system.	1 ☐	2 ☐	3 ☐	4 ☐	5 ☐	6 ☐	7 ☐	N/a ☐
9.The GUARDIAN system gave error messages that clearly told me how to fix problems.	1 ☐	2 ☐	3 ☐	4 ☐	5 ☐	6 ☐	7 ☐	N/a ☐

10. Whenever I made a mistake using the GUARDIAN system, I could recover easily and quickly.	1 ☐	2 ☐	3 ☐	4 ☐	5 ☐	6 ☐	7 ☐	N/a ☐
11. The information (such as online help, on-screen messages, and other documentation) provided with the GUARDIAN system was clear.	1 ☐	2 ☐	3 ☐	4 ☐	5 ☐	6 ☐	7 ☐	N/a ☐
12. It was easy to find the information I needed.	1 ☐	2 ☐	3 ☐	4 ☐	5 ☐	6 ☐	7 ☐	N/a ☐
13. The information was effective in helping me complete the tasks and scenarios.	1 ☐	2 ☐	3 ☐	4 ☐	5 ☐	6 ☐	7 ☐	N/a ☐
14.The organization of information on the GUARDIAN system screens was clear.	1 ☐	2 ☐	3 ☐	4 ☐	5 ☐	6 ☐	7 ☐	N/a ☐
Note: *The interface includes those items that you use to interact with the GUARDIAN system. For example, the language, buttons, text-boxes, etc.*								
15. The interface of the GUARDIAN system was pleasant.	1 ☐	2 ☐	3 ☐	4 ☐	5 ☐	6 ☐	7 ☐	N/a ☐
16. I liked using the interface of the GUARDIAN system.	1 ☐	2 ☐	3 ☐	4 ☐	5 ☐	6 ☐	7 ☐	N/a ☐
17. This GUARDIAN system has all the functions and capabilities I expect it to have.	1 ☐	2 ☐	3 ☐	4 ☐	5 ☐	6 ☐	7 ☐	N/a ☐
18. Overall, I am satisfied with the GUARDIAN system.	1 ☐	2 ☐	3 ☐	4 ☐	5 ☐	6 ☐	7 ☐	N/a ☐
19. List the most negative aspect(s) of the GUARDIAN system and/or interface:								

20. List the most positive aspect(s) of the GUARDIAN system and/or interface:	

2 Informal Carer

2.1 Information & recruitment session

Demographics					
Gender	☐ Male		☐ Female		☐ Other
Country FC lives:					
Education level	☐ Lower		☐ Medium		☐ Higher
Technology tools	Which of the following devices do you often (more than once a week) use?<br>I'm using a… (multiple answers possible)				
	☐ Computer		☐ Tablet		☐ Smartphone
Technology level	How do you rate your experience with technology (e.g. video calling, e-mailing, using whatsapp) on a scale of 1 (no experience) till 5 (much experience)?				
	1. None ☐	2. A bit ☐	3. Moderate ☐	4. Quite some ☐	5. Much experience ☐
Relationship	What is your relationship with the person you care for?				

	Partner<br>☐	Father/Mother<br>☐	Brother/Sister<br>☐	Friend/Acquaintance<br>☐	Other,<br>namely________<br>☐
Level of informal care	How often do you provide care to your loved one on average per week?				
	<1 day<br>☐	2- 3 days per week<br>☐	Daily<br>☐		
	How many years do you already provide cares/support to your loved one?				

2.2 Training and Equipment session at senior's home

**First Impression**	
Ask after introduction and demonstration of the Guardian system	
What is your first impression of the Guardian System?	
Is everything clear? What did you think of this information session?	
Schedule debriefing session	

2.3 Follow-up after 1 week

First impression & usability	
What is your first impression of the Guardian system after using it for a week?	
How often did you check the caregiver application? How did it go?	
Is Guardian easy to use on a daily basis? If so, why?	
Did you encounter any problems? If so, what problems and what did you do?	
Did you need support to use the system? If so, who helped you and how? Could you do it now by yourself?	

How do you perceive the interaction with the robot?<br>*(only if informal carer had interaction with the robot when being at the senior's home)*	
Do you have any other comments?	

2.4 Debriefing session - Interview

Qualitative questions: usability, willingness to pay, impact, ethics	
Give a short summary of what the participant said during the follow-up: *We spoke to each other last week and you said …*<br>*Was something different last week? How did it go then?*<br><br>How do you use the caregiver application? When do you use the caregiver application? Was there a time you got stuck using it?	
**Giving instructions**	
Describe your experience introducing Guardian to the senior?<br><br>*Did the senior had a lot of questions for you? What did you do to help?*	
**Setting up the system**	
How often and for what did you set up reminders and requests? How did it go?<br><br>What actions did you have difficulty with? What took a lot of time to do?	
**Dashboard**	
How often did you check the dashboard? What did you look at? Was the information clear? Would you like to monitor other things?	

Do you feel confident while using the Guardian system?	
Do you believe Guardian as you used it the last two weeks is useful in your daily life? Why?	
Which of the functionalities you tested did you find valuable/useful? And which not? Why? Do you miss anything?	
You have used Guardian for 2 weeks now, how was the interaction between you, the senior and the formal carer?<br><br>*Has anything changed? How did you experience that? Positive or negative?*	
Do you think you can adapt the system well enough to fit the personal situation (wishes and needs) of the senior?	

<table>
<tr><td>How do you expect your daily life would change when using GUARDIAN for a longer time?</td><td colspan="5"></td></tr>
<tr><td>How do you feel about your privacy when you come over to the senior’s home and the robot is standing there?
Do you feel that your privacy is sufficiently respected? Or how can this be improved?</td><td colspan="5"></td></tr>
<tr><td>Do you have any concerns about the impact of using Guardian on the senior’s interactions and relations with other people, like family and caregivers? Do you have any suggestions about how the social connectedness of the senior with other people can be improved through the robot?</td><td colspan="5"></td></tr>
<tr><td rowspan="2">What are you willing to pay for such a system as GUARDIAN? If the participant is not willing to pay: What’s stopping you from paying?</td><td>☐0,- € per month</td><td colspan="2">☐5,- € per month</td><td>☐10,- € per month</td><td>☐15,- € per month</td></tr>
<tr><td colspan="5">or I’m willing to pay __________ ,- € once</td></tr>
<tr><td>Read the statements to the participant and ask them to indicate how strongly they agree or disagree on a scale of 1 – 5.</td><td>1. Strongly Disagree</td><td>2. Disagree</td><td>3. Neutral</td><td>4. Agree</td><td>5. Strongly Agree</td></tr>
<tr><td>Using Guardian helps me to notice something is wrong in an early stage</td><td>☐</td><td>☐</td><td>☐</td><td>☐</td><td>☐</td></tr>
<tr><td>The Guardian system offers me peace of mind</td><td>☐</td><td>☐</td><td>☐</td><td>☐</td><td>☐</td></tr>
</table>

The Guardian system helps me to feel more involved in the care for my loved one	☐	☐	☐	☐	☐
GUARDIAN helps me to feel more equal in a conversation with a formal caregiver	☐	☐	☐	☐	☐
GUARDIAN strengthens the cooperation between caregivers	☐	☐	☐	☐	☐
Where your experiences using GUARDIAN in line with your expectations prior to the test? Why?					

2.5 Debriefing session – Questionnaires

Think about all the tasks that you have done with the GUARDIAN system while you answer the following questions. Please read each statement and indicate how strongly you agree or disagree with the statement by circling a number on the scale.

Note for researcher: You are allowed to use the opposite scale as well if that works better in your country but please make clear which scale is used.

TAM							
**Perceived Ease of Use (PE)**	**Completely disagree**	**Disagree**	**Somewhat disagree**	**Neutral**	**Somewhat agree**	**Agree**	**Completely agree**
I find Guardian system easy to use	1 ☐	2 ☐	3 ☐	4 ☐	5 ☐	6 ☐	7 ☐
Learning how to use technological tool is easy for me	1 ☐	2 ☐	3 ☐	4 ☐	5 ☐	6 ☐	7 ☐
It's easy to become skilful at using the Guardian system	1 ☐	2 ☐	3 ☐	4 ☐	5 ☐	6 ☐	7 ☐
**Perceived Usefulness (PU)**	**Completely disagree**	**Disagree**	**Somewhat disagree**	**Neutral**	**Somewhat agree**	**Agree**	**Completely agree**
Guardian system would improve my working performance	1 ☐	2 ☐	3 ☐	4 ☐	5 ☐	6 ☐	7 ☐
Guardian system would increase my productivity	1 ☐	2 ☐	3 ☐	4 ☐	5 ☐	6 ☐	7 ☐
Guardian system could make care work easier	1 ☐	2 ☐	3 ☐	4 ☐	5 ☐	6 ☐	7 ☐
**Attitude (AT)**	**Completely disagree**	**Disagree**	**Somewhat disagree**	**Neutral**	**Somewhat agree**	**Agree**	**Completely agree**
Working through Guardian system is a good idea	1 ☐	2 ☐	3 ☐	4 ☐	5 ☐	6 ☐	7 ☐

Working through Guardian system is a wise idea	1 ☐	2 ☐	3 ☐	4 ☐	5 ☐	6 ☐	7 ☐
I am positive towards Guardian system	1 ☐	2 ☐	3 ☐	4 ☐	5 ☐	6 ☐	7 ☐
**Behavioural intention (BI)**	**Completely disagree**	**Disagree**	**Somewhat disagree**	**Neutral**	**Somewhat agree**	**Agree**	**Completely agree**
I intend to check announcements from Guardian system frequently	1 ☐	2 ☐	3 ☐	4 ☐	5 ☐	6 ☐	7 ☐
I intend to be a heavy user of the Guardian system	1 ☐	2 ☐	3 ☐	4 ☐	5 ☐	6 ☐	7 ☐
**E-learning self-efficacy (SE)**	**Completely disagree**	**Disagree**	**Somewhat disagree**	**Neutral**	**Somewhat agree**	**Agree**	**Completely agree**
I feel confident finding information in the Guardian system	1 ☐	2 ☐	3 ☐	4 ☐	5 ☐	6 ☐	7 ☐

List the most negative aspect(s) of the GUARDIAN system and/or interface:	
List the most positive aspect(s) of the GUARDIAN system and/or interface:	

3 Formal Carer

3.1 Installation and Recruitment session

Demographics					
Gender	☐ Male	☐ Female		☐ Other	
Country FC lives:					
Education level	☐ Lower	☐ Medium		☐ Higher	
Work function					
Technology tools	Which of the following devices do you often (more than once a week) use?<br>I'm using a… (multiple answers possible)				
	☐ Computer	☐ Tablet		☐ Smartphone	
Technology level	How do you rate your experience with technology (e.g. video calling, e-mailing, using whatsapp) on a scale of 1 (no experience) till 5 (much experience)?				
	1. None<br>☐	2. A bit<br>☐	3. Moderate<br>☐	4. Quite some<br>☐	5. Much experience<br>☐
Experience	How many years experience do you have as a health care professional?				

3.2 Training and Equipment session at senior's home

First Impression	
Ask after introduction and demonstration of the Guardian system	
What is your first impression of the Guardian System?	
Is everything clear? What did you think of this information session?	
Schedule debriefing session	

3.3 Follow-up after 1 week

First impression & usability	
What is your first impression of the Guardian system after using it for a week?	
How often did you check the caregiver application?<br>*If we give them a list with tasks they can perform: Which tasks did you perform?* How did it go?	
Is Guardian easy to use on a daily basis? If so, why?	
Did you encounter any problems? If so, what problems and what did you do?	
Did you need support to use the system? If so, who helped you and how? Could you do it now by yourself?	

How do you perceive the interaction with the robot?<br>*(only if formal carer had interaction with the robot when being at the senior's home)*	
Do you have any other comments?	

3.4 Debriefing session – Interview

Qualitative questions: usability, willingness to pay, impact, ethics	
Give a short summary of what the participant said during the follow-up: *We spoke to each other last week and you said …* *Was something different last week? How did it go then?*<br><br>How do you use the caregiver application? When do you use the caregiver application? Was there a time you got stuck using it?	
**Giving instructions**	
Describe your experience introducing Guardian to the senior?<br><br>*Did the senior had a lot of questions for you? What did you do to help?*	
**Setting up the system**	
How often and for what did you set up reminders and requests? How did it go?<br><br>What actions did you have difficulty with? What took a lot of time to do?	
**Dashboard**	
How often did you check the dashboard? What did you look at? Was the information clear? Would you like to monitor other things?	

Do you feel confident while using the Guardian system?	
Do you believe Guardian as you used it the last two weeks is useful in your day-to-day job? Why?	
Which of the functionalities you tested did you find valuable/useful? And which not? Why? Do you miss anything?	
You have used Guardian for 2 weeks now, how was the interaction between you, the senior and the formal carer?<br><br>*Has anything changed? How did you experience that? Positive or negative?*	
Do you think you can adapt the system well enough to fit the personal situation (wishes and needs) of the senior?	

<table>
<tr><td>How do you expect your day-to-day job would change when using GUARDIAN for a longer time?</td><td colspan="5"></td></tr>
<tr><td>How do you feel about your privacy when you come over to the senior’s home and the robot is standing there?
Do you feel that your privacy is sufficiently respected? Or how can this be improved?</td><td colspan="5"></td></tr>
<tr><td>Do you have any concerns about the impact of using Guardian on the senior’s interactions and relations with other people, like family and caregivers? Do you have any suggestions about how the social connectedness of the senior with other people can be improved through the robot?</td><td colspan="5"></td></tr>
<tr><td rowspan="2">What are you willing to pay for such a system as GUARDIAN? If the participant is not willing to pay: What’s stopping you from paying?</td><td>☐0,- € per month</td><td>☐5,- € per month</td><td colspan="2">☐10,- € per month</td><td>☐15,- € per month</td></tr>
<tr><td colspan="5">or I’m willing to pay __________ ,- € once</td></tr>
<tr><td>Read the statements to the participant and ask them to indicate how strongly they agree or disagree on a scale of 1 – 5.</td><td>1. Strongly Disagree</td><td>2. Disagree</td><td>3. Neutral</td><td>4. Agree</td><td>5. Strongly Agree</td></tr>
<tr><td>Using Guardian helps me to notice something is wrong in an early stage</td><td>☐</td><td>☐</td><td>☐</td><td>☐</td><td>☐</td></tr>
<tr><td>GUARDIAN strengthens the cooperation between caregivers</td><td>☐</td><td>☐</td><td>☐</td><td>☐</td><td>☐</td></tr>
</table>

The Guardian system helps me to finetune the therapeutic plan	☐	☐	☐	☐	☐
The Guardian system helps me to save time	☐	☐	☐	☐	☐
The Guardian system gives me the feeling of a lower workload?	☐	☐	☐	☐	☐
Where your experiences using GUARDIAN in line with your expectations prior to the test? Why?					

3.5 Debriefing session - Questionnaires

Think about all the tasks that you have done with the GUARDIAN system while you answer the following questions. Please read each statement and indicate how strongly you agree or disagree with the statement by circling a number on the scale.

Note for researcher: You are allowed to use the opposite scale as well if that works better in your country but please make clear which scale is used.

TAM							
**Perceived Ease of Use (PE)**	**Completely disagree**	**Disagree**	**Somewhat disagree**	**Neutral**	**Somewhat agree**	**Agree**	**Completely agree**
I find Guardian system easy to use	1 ☐	2 ☐	3 ☐	4 ☐	5 ☐	6 ☐	7 ☐
Learning how to use technological tool is easy for me	1 ☐	2 ☐	3 ☐	4 ☐	5 ☐	6 ☐	7 ☐
It's easy to become skilful at using the Guardian system	1 ☐	2 ☐	3 ☐	4 ☐	5 ☐	6 ☐	7 ☐
**Perceived Usefulness (PU)**	**Completely disagree**	**Disagree**	**Somewhat disagree**	**Neutral**	**Somewhat agree**	**Agree**	**Completely agree**
Guardian system would improve my working performance	1 ☐	2 ☐	3 ☐	4 ☐	5 ☐	6 ☐	7 ☐
Guardian system would increase my productivity	1 ☐	2 ☐	3 ☐	4 ☐	5 ☐	6 ☐	7 ☐
Guardian system could make care work easier	1 ☐	2 ☐	3 ☐	4 ☐	5 ☐	6 ☐	7 ☐
**Attitude (AT)**	**Completely disagree**	**Disagree**	**Somewhat disagree**	**Neutral**	**Somewhat agree**	**Agree**	**Completely agree**
Working through Guardian system is a good idea	1 ☐	2 ☐	3 ☐	4 ☐	5 ☐	6 ☐	7 ☐

Working through Guardian system is a wise idea	1 ☐	2 ☐	3 ☐	4 ☐	5 ☐	6 ☐	7 ☐
I am positive towards Guardian system	1 ☐	2 ☐	3 ☐	4 ☐	5 ☐	6 ☐	7 ☐
**Behavioural intention (BI)**	**Completely disagree**	**Disagree**	**Somewhat disagree**	**Neutral**	**Somewhat agree**	**Agree**	**Completely agree**
I intend to check announcements from Guardian system frequently	1 ☐	2 ☐	3 ☐	4 ☐	5 ☐	6 ☐	7 ☐
I intend to be a heavy user of the Guardian system	1 ☐	2 ☐	3 ☐	4 ☐	5 ☐	6 ☐	7 ☐
**E-learning self-efficacy (SE)**	**Completely disagree**	**Disagree**	**Somewhat disagree**	**Neutral**	**Somewhat agree**	**Agree**	**Completely agree**
I feel confident finding information in the Guardian system	1 ☐	2 ☐	3 ☐	4 ☐	5 ☐	6 ☐	7 ☐

List the most negative aspect(s) of the GUARDIAN system and/or interface:	
List the most positive aspect(s) of the GUARDIAN system and/or interface:	
